# Aqueous Lumican Correlates with Central Retinal Thickness in Patients with Idiopathic Epiretinal Membrane: A Proteome Study

**DOI:** 10.1155/2022/9886846

**Published:** 2022-03-24

**Authors:** Wei-Cheng Chang, Cho-Hao Lee, Shih-Hwa Chiou, Chen-Chung Liao, Chao-Wen Cheng

**Affiliations:** ^1^Graduate Institute of Clinical Medicine, College of Medicine, Taipei Medical University, Taipei 11031, Taiwan; ^2^Department of Ophthalmology, Taoyuan General Hospital, Ministry of Health and Welfare, Taoyuan 33004, Taiwan; ^3^Division of Hematology and Oncology, Department of Internal Medicine, Tri-Service General Hospital, National Defense Medical Center, Taipei 114202, Taiwan; ^4^Department of Medical Research, Taipei Veterans General Hospital, Taipei 11217, Taiwan; ^5^School of Medicine, National Yang Ming Chiao Tung University, Taipei 11221, Taiwan; ^6^Institute of Pharmacology, National Yang Ming Chiao Tung University, Taipei 11221, Taiwan; ^7^Genomic Research Center, Academia Sinica, Taipei 11529, Taiwan; ^8^Metabolomics-Proteomics Research Center, National Yang Ming Chiao Tung University, Taipei 11221, Taiwan; ^9^Traditional Herbal Medicine Research Center, Taipei Medical University Hospital, Taipei Medical University, Taipei 11031, Taiwan; ^10^Cell Physiology and Molecular Image Research Center, Wan Fang Hospital, Taipei Medical University, Taipei 11031, Taiwan

## Abstract

Idiopathic epiretinal membrane (iERM) is a pathological fibrocellular change in the vitreoretinal junction over the macular area; however, possible pathogenic mechanisms remain unclear. Changes in the differential protein composition of the aqueous humor (AH) may represent potential molecular changes associated with iERM. To gain new insights into the molecular mechanisms of iERM pathology, a sensitive label-free proteomics analysis was performed to compare AH protein expressions in patients with cataracts with or without iERM. This study employed nanoflow ultra-high-performance liquid chromatography-tandem mass spectrometry to investigate protein compositions of the AH obtained from individual human cataract eyes from 10 patients with iERM and 10 age-matched controls without iERM. Eight proteins were differentially expressed between the iERM and control samples, among which six proteins were upregulated and two were downregulated. A gene ontology (GO) analysis revealed that iERM was closely associated with several biological processes, such as immunity interactions, cell proliferation, and extracellular matrix remodeling. Additionally, multiple proteins, including lumican, cyclin-dependent kinase 13, and collagen alpha-3(VI) chain, were correlated with the central retinal thickness, indicating a multifactorial response in the pathogenic process of iERM. Changes in the AH level of lumican between iERM and control samples were also confirmed by an enzyme-linked immunosorbent assay. In conclusion, several pathological pathways involved in iERM were identified in the AH by a proteomic analysis, including immune reactions, cell proliferation, and remodeling of the extracellular matrix. Lumican is a potential aqueous biomarker for predicting iERM development and monitoring its progression. More clinical parameters also need to be identified to complete the analysis, and those could provide additional targets for treating and preventing iERM.

## 1. Introduction

By definition, idiopathic epiretinal membrane (ERM; iERM) is a pathological proliferative fibrocellular change in the vitreoretinal junction over the macular area with no other ocular disease or secondary to any other intraocular surgery, intraocular disease, or ocular trauma [1]. It can reduce vision, cause metamorphopsia, and require vitreoretinal surgery to peel off the membrane [2]. Two main components, noncellular and cellular, of the ERM are often discussed as extracellular matrix (ECM) structures like fibronectin, vitronectin, and collagen and various cell types of retinal and extraretinal origin, such as glial cells, astrocytes, Muller cells, retinal pigment epithelial cells, and myofibroblasts [3–8]. Some studies also reported that specific cytokines are expressed by iERMs, such as vascular endothelial growth factor, interleukin- (IL-) 6, transforming growth factor-beta, and connective tissue growth factor [9–11]. Although the true pathogenic mechanisms are not well known, changes in differential protein compositions are associated with iERM.

A proteomics analysis is a valuable approach to define changes in protein levels in tissues and cells. It has been used to examine biofluids such as the aqueous humor (AH), vitreous humor (VH), tears, and serum to find many biomarkers in diabetic retinopathies [12]. The emergence of liquid biopsies for diabetic retinopathies and other vitreoretinal diseases was also noted for precision medicine [13, 14]. Recent proteomics studies of human AH revealed many proteins in patients with intraocular disease [13, 15–19]. Pollreisz et al. [20] first performed isobaric tags for relative and absolute quantitation (iTRAQ) 4-plex quantitative protein analysis of the aqueous and vitreous fluids from human eyes with iERM. They found 323 proteins in the aqueous and vitreous fluids from eyes with iERM, and only 3.96% of the identified proteins exhibited significant differential expression between the aqueous and vitreous fluids. Yu et al. [21] first examined the vitreous proteomes from iERMs and donor controls in a differential analysis by reversed-phase high-performance liquid chromatography. It revealed 226 significant protein changes in the vitreous proteome of iERM patients compared to the vitreous of normal control subjects. Mandal et al. [22] analyzed vitreous samples from patients with iERM and idiopathic macular hole (iMH) with two-dimensional polyacrylamide gel electrophoresis (2D-PAGE), and Christakopoulos et al. [23] compared iERM samples to internal limiting membrane samples from patients with iMH with label-free quantitative nanoflow-liquid chromatography-tandem mass spectrometry (n-LC-MS/MS). However, until now, no study has reported differential changes in the protein composition of the AH from individual patients and controls with iERM.

So far, there are no proteomics studies that focused on analyzing aqueous fluids from eyes with iERM without a pooling method. In this study, we employed nanoflow ultra-high-performance (UHP) LC-MS/MS to investigate the protein composition of the aqueous obtained from individual human cataract eyes with iERM and aged-matched controls. This sensitive proteomics approach should help elucidate the underlying pathophysiology of iERM disease using a relatively small amount of aqueous sample and thus was the favored methodological approach selected for this investigation. This research may reveal valuable insights into molecular changes in the AH of patients with iERM.

## 2. Materials and Methods

### 2.1. Subjects

**Figure 1:**
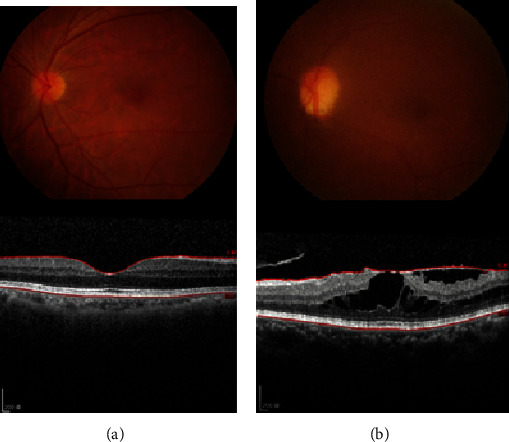
Optical coherence tomography (OCT) of (a) a healthy retina and (b) an idiopathic epiretinal membrane (ERM; iERM). The cross-sectional B-scan was obtained with a spectral-domain OCT device in (a) a healthy eye and (b) an eye with iERM. (b) shows the retina of an eye with an ERM.

The Medical Ethics and the Institutional Review Board of Taoyuan General Hospital (TYGH), Ministry of Health and Welfare, approved the study protocol (TYGH109009), and the study was conducted according to the tenets of the *Declaration of Helsinki*. All study participants provided written informed consent before their enrollment, and the nature and possible consequences of the study were fully explained to them. Human AH samples from treatment-naive patients with iERM (*n* = 10) and age-matched controls (*n* = 10) were collected when the subjects were undergoing cataract surgery at TYGH (Taoyuan, Taiwan). The diagnostic criterion for iERM was defined by optical coherence tomography (OCT) without other ocular diseases, trauma, or a previous intraocular operative history. Control eyes belonged to senile cataract patients free from other ocular or systemic diseases. In both groups, the inclusion criteria were cataract patients aged older than 55 years. The exclusion criteria were a history of any systemic disease including hypothyroidism or ocular disorders including glaucoma, previous ocular infectious diseases, retinal vascular disorders, ocular surgery, or trauma. The best-corrected visual acuity was measured as the logarithm of the minimum angle of resolution (logMAR). Spectral-domain- (SD-) OCT (Heidelberg Engineering, Germany) was used for the OCT examination (Figure 1). The central retinal thickness was measured with a caliper tool of Heidelberg OCT software.

### 2.2. AH Sample Collection

AH samples were collected from patients during the implantation of phakic intraocular lenses. The sample was collected using a 1 ml tuberculin syringe with a 30-gauge needle at the limbus under a surgical microscope before any other entry into the eye to avoid hemorrhaging and other ocular surface contaminants. Approximately 50~100 *μ*L of AH was collected from each patient by anterior chamber paracentesis. Undiluted AH samples were collected and stored at −80°C until preparation was initiated within 24 h.

### 2.3. n-UPLC-MS/MS

Protein concentrations of AH samples were determined by a dye-binding method based on the Bradford assay (Bio-Rad Laboratories, Richmond, CA, USA) (Table 1), and samples were further diluted in 1x phosphate-buffered saline to a final concentration of 0.1 *μ*g/*μ*L. Samples were prepared according to the SMART digestion kit protocol from Thermo Fisher Scientific (Waltham, MA, USA) and cleaned up by solid-phase extraction plates from Thermo Fisher Scientific. The resulting peptides collected from the filters were dried in a vacuum centrifuge and stored at −80°C. Thereafter, 50 *μ*L of diluted AH samples was resuspended in 0.1% formic acid and analyzed by n-UPLC-MS/MS. Tryptic peptides were loaded into an Elite mass spectrometer with a nanoelectrospray ionization source (Thermo Electron, MA, USA) connected to a nanoACQUITY UPLC system (Waters, MA, USA). Peptide samples were separated by a 25 cm × 75 *μ*m BEH130 C18 column (Waters) with a 0%~95% segmented gradient of 3%~40% B for 168 min, 40%~95% B for 2 min, and 95% B for 10 min at a flow rate of 0.5 *μ*L/min. Mobile phase A was 0.1% formic acid in water, while mobile phase B was 0.1% formic acid in acetonitrile. The mass spectrometer was based on the data-dependent acquisition method (isolation width: 1.5 Da). According to the data-dependent acquisition method, the first ten most intensively charged peptide ions were selected and fragmented by the collision-induced dissociation method.

### 2.4. Protein Identification

The acquired MS/MS raw data files were then applied to search against a UniProt human protein database (containing 20,387 protein sequences; released April 2021) with PEAKS Studio 7.5 (Bioinformatic Solution, Ontario, CA). PEAKS Studio 7.5 was combined with UniProt's protein database search settings as follows: the enzyme was set to trypsin; it is up to two missing cutting sites; precursor and fragment mass tolerances were 20 ppm and 0.8 Da, respectively; and the false discovery rate (FDR) was <1%, as obtained from a search of the decoy database. Furthermore, each identified protein had to contain at least one unique peptide, and the protein quantification method was based on a label-free quantitative analysis. Additionally, spectral counts were normalized to the total identification spectrum of each biological sample.

### 2.5. Enzyme-Linked Immunosorbent Assay (ELISA)

According to the manufacturer's instructions, the ELISA was performed to measure lumican concentrations in iERM and control AH samples with a Human Lumican ELISA Kit (EH310RB, Thermo Fisher Scientific).

### 2.6. Statistical Analysis

Clinical data were analyzed using Stata (vers. 16.1, StataCorp, College Station, TX, USA) to define the statistical significance between groups by a *t*-test or chi-squared test, and *p* < 0.05 was considered statistically significant. Statistical analysis by Fisher's exact test was used to verify that there was no statistically significant difference in age between the iERM and age-matched control groups (Table 1).

## 3. Results

Demographic data of the iERM and control groups are shown in Table 1 and Table S1. The mean age of patients with iERM was 74.00 ± 5.23 years, and that of control individuals was 73.90 ± 5.72 years. All patients with iERM revealed an ERM by an OCT examination. The mean protein concentration of the AH was 0.36 ± 0.16 *μ*g/*μ*L in the iERM group and 0.22 ± 0.06 *μ*g/*μ*L in the control group. There were statistical differences between total protein contents in these groups (*p* = 0.029; Fisher's exact test, Wilcoxon test, or Kruskal-Wallis test) and no statistical differences between ages in these groups (*p* = 0.675; Fisher's exact test, Wilcoxon test, or Kruskal-Wallis test).

In total, 405 proteins were successfully identified by LC-ESI MS/MS in iERM and control AH samples (Fig. S1). Compared to the control proteome, 189 proteins were present at higher levels, and 216 proteins were present at lower levels in the iERM proteome. To understand the biological meanings of the changes in protein expressions observed in iERM, differentially expressed proteins were analyzed in terms of “molecular functions,” “biological processes,” and “cellular components” by gene ontology (GO) annotations. Our results indicated that differentially expressed proteins in iERM and controls were associated with molecular functions, biological processes, and cellular components. The most important biological processes for downregulated proteins were adaptive immune responses, carbohydrate catabolic processes, glycolipid catabolic processes, and glycoside catabolic processes. In contrast, biological processes of upregulated proteins were strongly associated with ECM organization, negative regulation of endopeptidase activity, positive regulation of cell proliferation, negative regulation of cell proliferation, axon guidance, and cell adhesion. In terms of molecular functions, results revealed that collagen binding, ECM structural constituents, serine-type endopeptidase inhibitor activity, ATP binding, and cyclin binding were elevated. In contrast, antigen binding, alpha-N-acetylgalactosaminidase activity, alpha-galactosidase activity, and protein homodimerization activity showed decreased levels.

We used the Ingenuity Pathway Analysis (IPA, Qiagen) to show canonical pathways that were potentially involved in iERM pathogenesis.  Table 2 lists pathways that were associated with AH proteins from patients with iERM and the controls. The top canonical pathways, included interleukin- (IL-) 15 signaling, B-cell receptor signaling, systemic lupus erythematosus in the B-cell signaling pathway, and communication between innate and adaptive immune cells, and significant associations with AH proteins were demonstrated.

A statistical analysis was performed on these 405 proteins, and eight proteins statistically significantly differed in content in iERM samples compared to the controls (Table 3). Among these eight proteins whose contents had changed, six proteins were higher in the iERM group, including lumican, thyroxine-binding globulin (TBG), double-stranded (ds)RNA-specific editase 1, cyclin-dependent kinase 13, collagen alpha-3(VI) chain, and cytoplasmic transfer (t)RNA 2-thiolation protein 1. Another two proteins, viz., immunoglobulin (Ig) heavy variable 5-10-1 and alpha-N-acetylgalactosaminidase, exhibited lower contents in the iERM group (Table 3).

**Figure 2:**
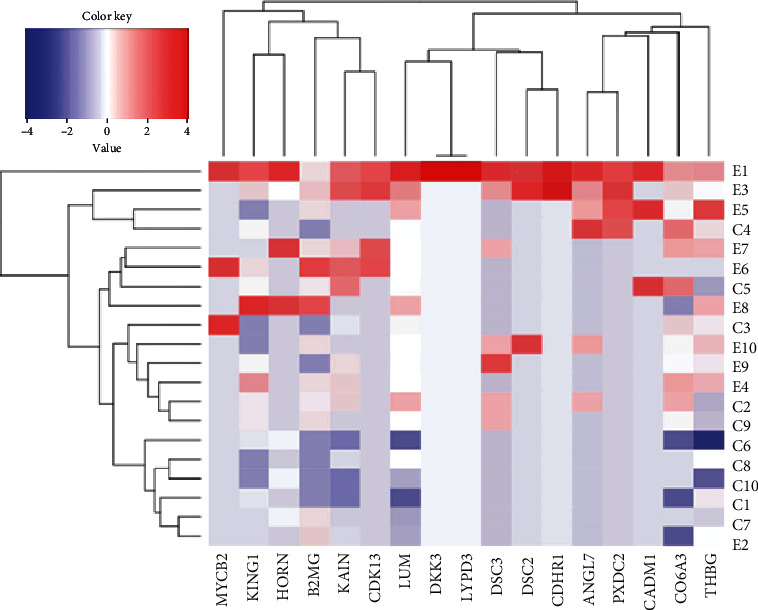
Heat map generated from an R software analysis with the average relative intensity reference. Numbers E1~E10 represent patients with idiopathic epiretinal membrane (iERM), and C1~C10 represent controls. They rank in numbers with the central retinal thickness (CRT) from thick to thin.

**Figure 3:**
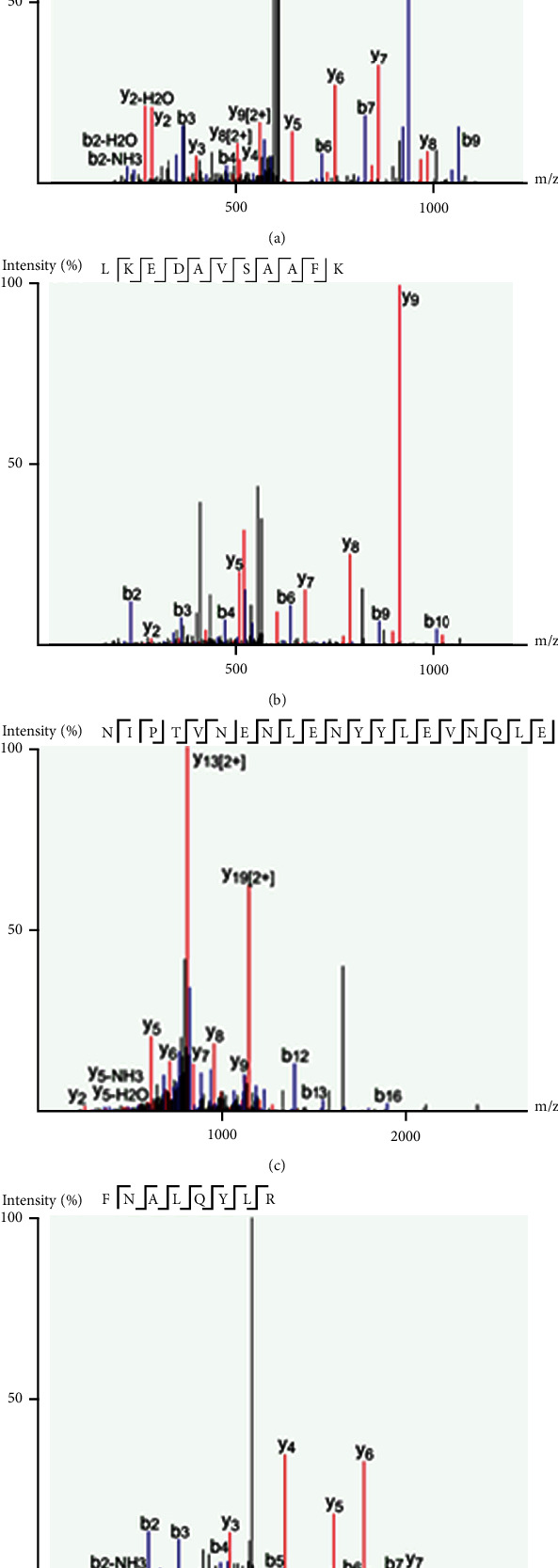
Lumican peptide matching sequences.

**Figure 4:**
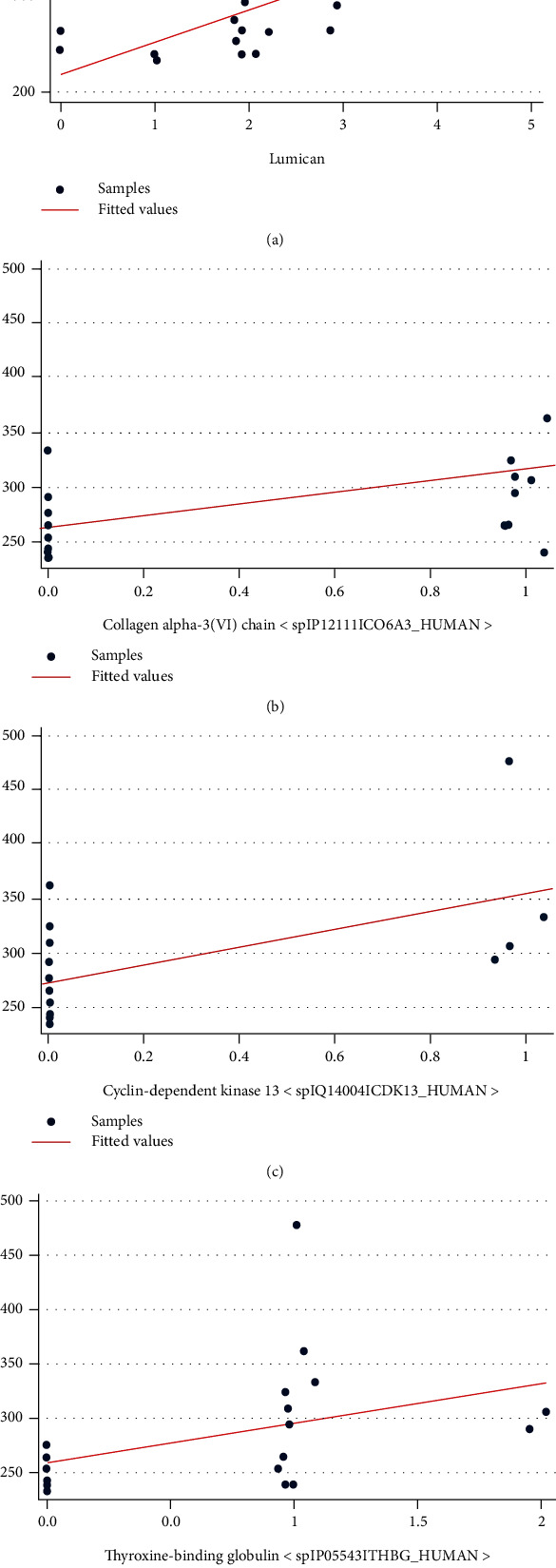
Correlations between aqueous proteins and the central retinal thickness (CRT). Vertical values represent the CRT (*μ*m) measured by OCT. Horizontal values denote the contents of the proteins measured by mass spectrometry (spectral count (SpC)). Correlations were calculated as Pearson's correlation coefficients (*r*). Proteins were significantly positively correlated with the CRT (a–d).

**Figure 5:**
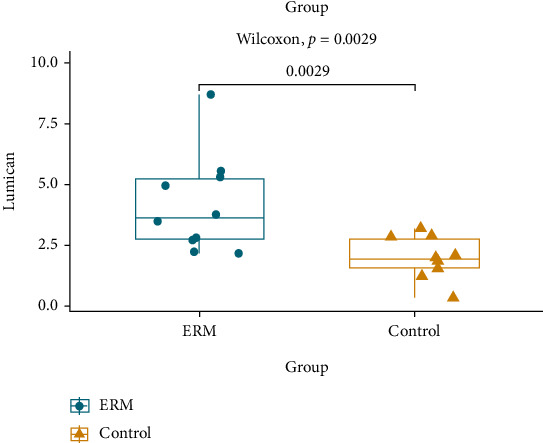
ELISA analysis revealed significant concentration changes between the idiopathic epiretinal membrane (iERM) and control groups. Vertical values denote the concentration of lumican (*μ*g/mL). The average concentration of lumican was significantly elevated in patients with iERM (4.172 *μ*g/mL) compared to the control group (2.006 *μ*g/mL) (*p* = 0.0058).

Among the 405 proteins, 17 proteins were positively correlated with the central retina thickness (CRT) (Table 4, Figure 2). Proteins that were positively correlated with the CRT, which significantly differed in iERM samples compared to the controls, included lumican, TBG, cyclin-dependent kinase (CDK) 13, and collagen alpha-3(VI) chain (Figure 3). Additionally, the proteomics analysis revealed an increased level of lumican in iERM samples compared to the controls with the most significant correlation to the CRT among the eight proteins. The peptide sequence of lumican is shown in Figure 4. Associations and relative levels of lumican and the other seven major proteins are also shown in Figure S2. We then chose lumican for further ELISA verification (multiple of change = 1.70, *p* = 0.023) (Table 4; Figure 3). We performed an ELISA to determine the concentration of lumican, and the average concentration of lumican was significantly (*p* = 0.0058) elevated in patients with iERM (4.172 *μ*g/mL) compared to control subjects (2.006 *μ*g/mL) (Figure 5).

## 4. Discussion

This is the first study to use label-free nanoflow UHP LC-MS/MS quantitation to analyze the human AH proteome in iERM disease compared to controls. We found increased total protein levels in the AH and changes in the AH protein profile in iERM subjects, which differed from controls. Eight aqueous proteins were significantly higher in the AH of patients with iERM. Notably, lumican was positively correlated with the CRT, which is a useful measurement of iERM progression. ELISA confirmation of the evaluation of aqueous lumican levels further validated the proteomics findings. The increase in the AH lumican concentration may reflect an increased risk of pathogenic features of iERM. However, we have a limited understanding of the exact molecular events that regulate these eight significantly changed protein expressions in retinal disease.

Relationships between proteome parameters and eye diseases were previously studied. A similar technique was recently used to analyze changes in the AH of patients with keratoconus (KC) and patients with myopia [24, 25]. Yu et al. [21] performed similar research analyzing the vitreous proteome of patients with iERM and healthy donor controls and concluded that iERM involves a complicated pathological process including inflammation, immune responses, and cytoskeletal remodeling, with similar results as this study. The proteome of the AH was proven to be closely associated with fundal diseases [19, 20]. Although the aqueous protein does not come into direct contact with the retina, the ECM and secreted products may leak into intraocular fluids of the eye. Pollreisz et al. showed that only 3.96% of identified proteins exhibited significant differential expression between the aqueous and vitreous fluids from human eyes with iERM [20].

Furthermore, this leakage can increase the total protein level in bodily fluids. The increase in the total protein concentration in fluids can be used as a marker of the severity of fibrocellular changes. Similarly, increases in protein levels were observed in both the VH and AH in patients with diabetes mellitus retinopathy [16, 26, 27]. Our data showed that total protein levels in the AH were higher in iERM patients. Both the iERM and control groups had the same background conditions of cataracts; thus, we assumed that dysregulated proteins we found in the AH were related to the iERM pathogenesis. This finding assumes that elevated total protein levels may also serve as a biomarker for pathogenic changes in iERM.

Lumican is a keratan sulfate proteoglycan widely expressed in connective tissues, and it is critical in maintaining corneal transparency [28]. It is also a component of the sclera, and thinning of the sclera was found in *Lum*^−*/*−^ knockout mice [29]. A proteomics study of postmortem human eyes revealed that lumican was also found in Bruch's membrane, the choroid, and neurosensory retinae [30], where it was highly associated with ERM's origin. Due to being a member of the class II small leucine-rich proteoglycan (SLRP) family, lumican can maintain retinal homeostasis, is involved in collagen fibrillogenesis, and regulates proinflammatory responses [31]. Animal studies also proved increased expression of lumican along with age and modifications of lumican by macrophages, neutrophils, and polymorphonuclear neutrophils in immune responses [32–34]. A possible pathogenesis of iERM was shown by Bu et al. to be associated with age-related accumulation of advanced glycation end products which possibly contributes to anomalous posterior vitreous detachment. Remodeling of the ECM at the vitreoretinal interface by aging and fibrotic changes contributes to iERM pathogenesis [35]. ERM was postulated to involve the proliferation of fibroblasts, glial cells, and astrocytes after ILM disruption, and ECM structures where lumican is located and in which it is involved were discussed. Extracellular microstructures formed by cytoskeletal and ECM proteins contribute to maintaining retinal integrity and visual functions [36–38]. Genetic studies of gene polymorphisms in humans also support a role for lumican in scleral involvement of myopia which is closely associated with fibromodulin [39–42]. Alterations in lumican may thus contribute to myopia and various retinal diseases. Another recent study showed that lumican was negatively associated with mean defect (MD) values of a visual field test in patients with glaucoma, influencing the aqueous outflow resistance by trabecular meshwork reformation [39]. Considering the hypothesis of lumican's location and close association with ERM pathogenesis, all previous studies provided hints that lumican may contribute to iERM formation with scleral and ECM involvement and to maintaining the ocular immunologic status in iERM. Its expression may be identified as one of the hallmarks for ERM.

Furthermore, the increase in lumican levels in patients with iERM was also associated with an advanced status of iERM. Our finding of significant changes in lumican expression indicates that it could serve as a potential biomarker in managing iERM; however, further investigation is needed to elucidate the roles of lumican in the iERM pathogenesis. Another microstructural protein, collagen alpha-3(VI) chain, binds ECM proteins that aid in microfibril formation [40]. It was associated with diseases related to muscles and connective tissues [41, 42]. The appearance of this structural protein in the AH may indicate a possible loss of integrity of the retina.

Levels of three cell proliferation-related proteins, dsRNA-specific editase 1, CDK 13, and cytoplasmic tRNA 2-thiolation protein 1, were altered in the aqueous of patients with iERM. dsRNA-specific editase 1 catalyzes the hydrolytic deamination of adenosine to inosine in dsRNA [43] in nucleoli. It is expressed by the *ADARB1* gene, which is highly expressed in the brain and heart [44]. It was reported to be involved in neurodevelopmental disorders of hypotonia, microcephaly, and seizures (NEDHYMS) [45]. CDK 13 is responsible for RNA splicing [46] in nucleoli and is highly expressed in the fetal brain, liver, muscles, and adult brain. Dysregulation of CDK 13 is a factor leading to congenital heart defects, dysmorphic facial features, and intellectual developmental disorder (CHDFIDD) [47]. Cytoplasmic tRNA 2-thiolation protein 1 directly binds tRNAs and probably acts by catalyzing adenylation of tRNAs, an intermediate step required for 2-thiolation in the cytoplasm [48]. In terms of the eye, overexpressions of dsRNA-specific editase 1, CDK 13, and cytoplasmic tRNA 2-thiolation protein 1 are unknown in retinal disease based on past studies. We suspect that these three proteins are involved in cell proliferation processes in the formation of fibroproliferative iERM. This finding related to iERM may provide new investigation targets for future research.

TBG is well known in the serum and binds thyroid hormones in the blood circulation to regulate thyroid function. Upregulation of TBG leads to more binding of the thyroid hormone, which decreases the amount of free hormone available in the blood. This leads to stimulation of the thyroid-stimulating hormone and the production of more thyroid hormone. Upregulation of TBG levels may be caused by hypothyroidism, liver disease, pregnancy, acute intermittent porphyria, or genetics [49]. In this study, a significant change in TBG was found in patients with iERM; however, we did not determine serum levels in these patients with iERM. Further surveys can be arranged to compare serum and aqueous fluid TBG levels in concert with TBG or even a thyroid function test, which could be an early diagnostic biomarker.

Two downregulated proteins, Ig heavy variable 5-10-1 and alpha-N-acetylgalactosaminidase, were also found in aqueous changes in patients with iERM. Alpha-N-acetylgalactosaminidase is an isoenzyme of alpha-galactosidases that functions in the lysosome [50] and is related to Schindler disease [51] and Kanzaki disease [52] with variable neuroaxonal dystrophy and neurological signs. Therefore, we considered lysosome function to be involved in the iERM formation process. Ig heavy variable 5-10-1 is in the V region of the variable domain of Ig heavy chains that participate in antigen recognition [53]. Additionally, B lymphocytes secrete Igs, also known as antibodies that mediate the effector phase of humoral immunity [54]. Recently, some research showed significant changes in Igs in tear protein profiles in specific diseases like diabetic retinopathy, primary open-angle glaucoma, and postrefractive surgery [55, 56]. In our study, we found a decrease in Ig heavy variable 5-10-1 in the iERM group. This indicated some immune interactions in iERM formation. These findings provide us with more information on the pathogenic process of iERM formation.

In this study, we investigated the protein composition of the AH obtained from human cataract eyes with iERM and aged-matched controls without iERM. To the best of our knowledge, this is the first study to individually examine differential changes in the protein composition of the AH in iERM. However, several limitations should be specified. First, only 10 samples in each group were investigated, and future larger-scale studies could help verify our results. Second, only a small amount of AH (50~100 *μ*L) could be obtained due to anatomical features, which limited our ability to conduct subsequent validation assays. Third, the development of multiplex immunoassays can be improved. Finally, we can only provide associations of proteomic data with clinical presentations due to a lack of essential evidentiary support. Thus, the exact pathway by which lumican is involved in the iERM pathogenesis remains unknown. More studies to analyze the lumican levels in iERM patients are needed, and further serum and vitreous analyses of iERM patients should be conducted. More future investigations of molecular pathways are also needed to discuss how and why the proteomics data varied with the CRT and ultimately supply better knowledge of iERM for the whole of humanity.

## 5. Conclusions

In conclusion, iERM involves a complicated pathological process, including several proteins that participate in immune reactions, cell proliferation, and remodeling of the ECM, which were identified in the AH by a proteomics analysis. Thus, lumican could be a potential aqueous biomarker for predicting iERM development and monitoring its progression. However, more clinical parameters also need to be identified for a more-comprehensive analysis and to provide additional targets for treating and preventing iERM.

## Figures and Tables

**Table 1:** Samples for the proteomic analysis.

Variable	Control group	ERM group	*p* value
ERM	254.20 ± 12.97	319.50 ± 65.70	0.004^#^
Gender			0.007
Female	8 (80.0%)	2 (20.0%)	
Male	2 (20.0%)	8 (80.0%)	
Age	73.90 ± 5.72	74.00 ± 5.23	0.675^#^
VA	0.42 ± 0.10	0.29 ± 0.20	0.128^#^
LogMAR	0.39 ± 0.11	0.79 ± 0.67	0.128^#^
AXL	23.83 ± 1.17	24.26 ± 2.01	0.631^#^
Protein concentration	0.22 ± 0.06	0.36 ± 0.16	0.029^#^
Side			1.000^#^
Left	4 (40.0%)	5 (50.0%)	
Right	6 (60.0%)	5 (50.0%)	

#Analyzed by Fisher's exact test. ^∗^Data are expressed as the mean ± standard deviation. ERM: epiretinal membrane; VA: visual acuity; MAR: minimal angle of resolution; AXL: axial length.

**Table 2:** Pathway analysis of aqueous humor (AH) proteins using Ingenuity Pathway Analysis tools.

Canonical pathway	Overlapping proteins in the iERM and control groups
Interleukin-15 signaling	3
B-cell receptor signaling	3
Systemic lupus erythematosus in B-cell signaling pathway	3
Communication between innate and adaptive immune cells	3
Complement system	1

**Table 3:** Statistically significantly regulated proteins in patients with epiretinal membrane (ERM) versus controls in a proteomics analysis.

Protein ID	Protein name	Gene name	ERM group	Control group	*p* value	Multiple of change ERM/control
P51884	Lumican	LUM	2.51 ± 1.19	1.47 ± 0.84	0.023^#^	1.70748299
P05543	Thyroxine-binding globulin	THBG	1.10 ± 0.57	0.39 ± 0.50	0.004^#^	2.82051282
A0A0J9	Immunoglobulin heavy variable 5-10-1	HV5X1	0.11 ± 0.34	1.05 ± 1.23	0.027^#^	0.1047619
P17050	Alpha-N-acetylgalactosaminidase	NAGAB	0.00 ± 0.00	0.38 ± 0.48	0.031^#^	NA
P78563	Double-stranded RNA-specific editase 1	RED1	0.39 ± 0.51	0.00 ± 0.00	0.031^#^	NA
Q14004	Cyclin-dependent kinase 13	CDK13	0.41 ± 0.53	0.00 ± 0.00	0.031^#^	NA
P12111	Collagen alpha-3(VI) chain	CO6A3	0.70 ± 0.48	0.20 ± 0.42	0.039^#^	3.5
Q7Z7A3	Cytoplasmic tRNA 2-thiolation protein 1	Q7Z7A3	0.52 ± 0.54	0.10 ± 0.32	0.045^#^	5.2

^#^Tested by Fisher's exact test.

**Table 4:** Correlations between proteomics data and the central retinal thickness.

Protein ID	Protein name	Gene name	Correlation, *r*	*p* value
P51884	Lumican^#^	*LUM*	0.65573455	0.002
P01042	Kininogen-1	*KNG1*	0.50337911	0.024
P05543	Thyroxine-binding globulin^#^	*THBG*	0.38883989	0.049
Q86YZ3	Hornerin	*HORN*	0.5426932	0.013
Q02487	Desmocollin-2	*DSC2*	0.54640969	0.013
P61769	Beta-2-microglobulin	*B2MG*	0.48090844	0.032
P12111	Collagen alpha-3(VI) chain^#^	*CO6A3*	0.12831005	0.032
Q6UX71	Plexin domain-containing protein 2	*PXDC2*	0.52355015	0.018
Q14574	Desmocollin-3	*DSC3*	0.46152818	0.041
P29622	Kallistatin	*KAIN*	0.55527954	0.011
O43827	Angiopoietin-related protein 7	*ANGL7*	0.48941527	0.029
Q9BY67	Cell adhesion molecule 1	*CADM1*	0.47306149	0.035
Q14004	Cyclin-dependent kinase 13^#^	*CDK13*	0.59142594	0.006
O75592	E3 ubiquitin-protein ligase MYCBP2	*MYCB2*	0.44938655	0.047
Q9UBP4	Dickkopf-related protein 3	*DKK3*	0.78550025	0.001
Q96JP9	Cadherin-related family member 1	*CDHR1*	0.6932842	0.001
O95274	Ly6/PLAUR domain-containing protein 3	*LYPD3*	0.78550025	0.0001

Tested by Pearson's correlation coefficients. ^#^Proteins statistically significantly differentially regulated in epiretinal membrane patients.

## Data Availability

All data generated or analyzed during this study are available from the corresponding author on reasonable request.

## Abbreviations

AH: Aqueous humor
AXL: Axial length
CDK: Cyclin-dependent kinase
CRT: Central retinal thickness
ECM: Extracellular matrix
ELISA: Enzyme-linked immunosorbent assay
ERM: Epiretinal membrane
GO: Gene ontology
iERM: Idiopathic epiretinal membrane
Ig: Immunoglobulin
IPA: Ingenuity Pathway Analysis
LASIK: Laser-assisted in situ keratomileusis
MAR: Minimal angle of resolution
MD: Mean defect
OCT: Optical coherence tomography
RNA: Ribonucleic acid
SMILE: Small incision lenticule extraction
TBG: Thyroxine-binding globulin
VA: Visual acuity
VH: Vitreous humor.

## Supplementary Materials

Supplementary Materials Table S1: demographic characteristics of enrolled patients. Fig. S1: protein expression distributions of the idiopathic epiretinal membrane (iERM) and control groups. In total, 405 proteins were identified by LC-ESI MS/MS in iERM and control aqueous humor (AH) samples; 344 proteins were detected in the iERM group, and 350 proteins were detected in the control group. Fig. S2: relative levels of lumican and its associations with other major proteins.
