# Supplementary material for: Aqueous Lumican Correlates with Central Retinal Thickness in Patients with Idiopathic Epiretinal Membrane: A Proteome Study
Source: Dis Markers. 2022 Mar 24;2022:9886846. doi: 10.1155/2022/9886846 (PMC9106516; doi:10.1155/2022/9886846)
Supplement: Supplementary Materials — Table S1: demographic characteristics of enrolled patients. Fig. S1: protein expression distributions of the idiopathic epiretinal membrane (iERM) and control groups. In total, 405 proteins were identified by LC-ESI MS/MS in iERM and control aqueous humor (AH) samples; 344 proteins were detected in the iERM group, and 350 proteins were detected in the control group. Fig. S2: relative levels of lumican and its associations with other major proteins. [file 9886846.f1.docx]

# Supplementary Information

**S1 Table. Demographic characteristics of enrolled patients**

| **case** | **Age** | **Gender** | **Protein concentration(μg/μl)** | **VA(Snellen Chart)** | **Axial length(um)** | **Central retinal thickness(um)** |
| --- | --- | --- | --- | --- | --- | --- |
| E1 | 81 | M | 0.544 | 0.3 | 24.11 | 291 |
| E2 | 74 | M | 0.365 | 0.3 | 23.14 | 333 |
| E3 | 77 | M | 0.184 | 0.05 | 28.76 | 309 |
| E4 | 67 | M | 0.604 | 0.5 | 25.3 | 306 |
| E5 | 82 | M | 0.557 | 0.1 | 23.57 | 362 |
| E6 | 72 | M | 0.296 | 0.6 | 23.97 | 294 |
| E7 | 72 | M | 0.248 | 0.5 | 23.72 | 265 |
| E8 | 67 | F | 0.204 | 0.05 | 21.85 | 477 |
| E9 | 71 | F | 0.344 | 0.3 | 22.21 | 234 |
| E10 | 77 | M | 0.223 | 0.3 | 25.99 | 324 |
| C1 | 69 | F | 0.176 | 0.4 | 23.33 | 244 |
| C2 | 69 | F | 0.237 | 0.5 | 22.97 | 240 |
| C3 | 77 | F | 0.157 | 0.4 | 26.77 | 254 |
| C4 | 71 | F | 0.139 | 0.3 | 23.7 | 276 |
| C5 | 81 | M | 0.3 | 0.5 | 24.26 | 264 |
| C6 | 71 | F | 0.197 | 0.5 | 23.02 | 265 |
| C7 | 86 | F | 0.229 | 0.4 | 23.18 | 240 |
| C8 | 70 | F | 0.171 | 0.6 | 23.67 | 254 |
| C9 | 74 | F | 0.274 | 0.3 | 24.49 | 237 |
| C10 | 71 | M | 0.305 | 0.3 | 22.88 | 265 |

**Fig. S1. Protein expression distributions of the idiopathic epiretinal membrane (iERM) and control groups**

In total, 405 proteins were identified by LC-ESI MS/MS in iERM and control aqueous humor (AH) samples; 344 proteins were detected in the iERM group and 350 proteins were detected in the control group.

**
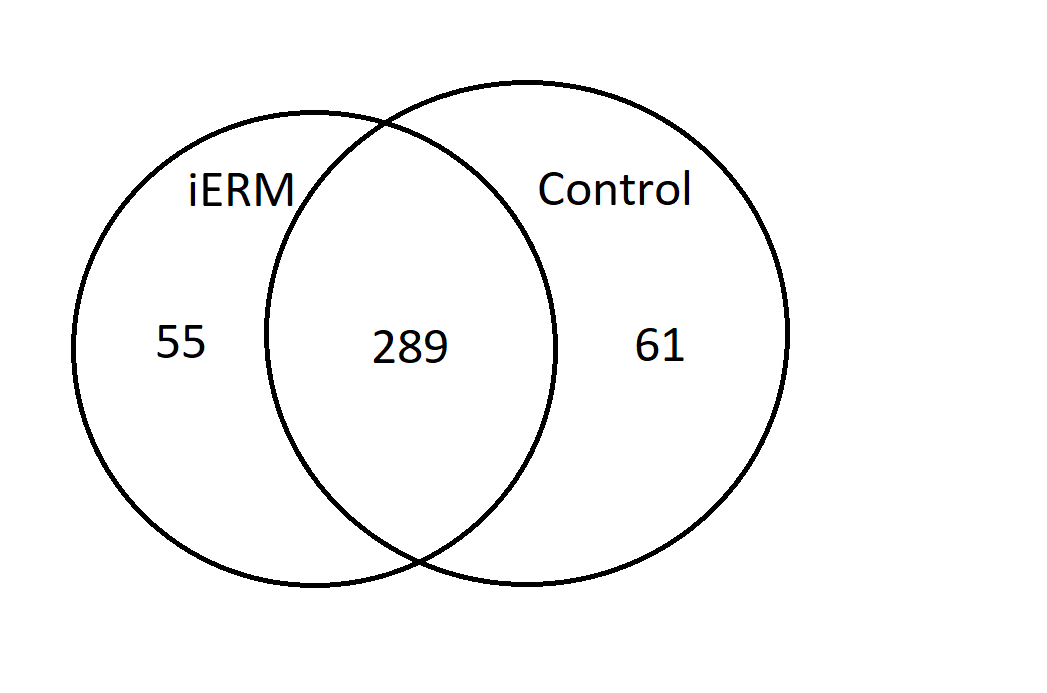
**

**Fig. S2. Relative levels of lumican and its associations with other major proteins.**

**
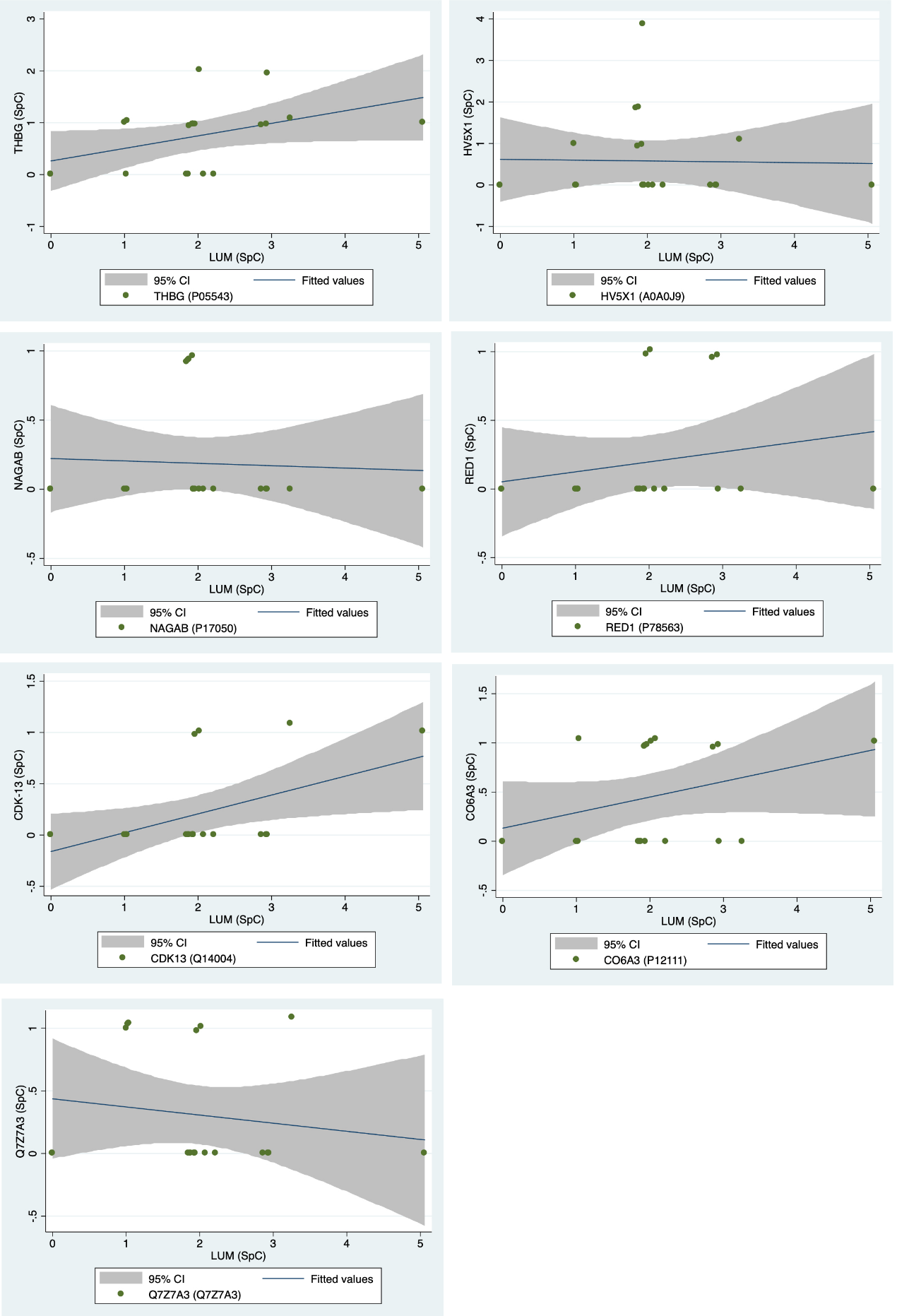
**
